# Automatic extraction, prioritization and analysis of gut microbial metabolites from biomedical literature

**DOI:** 10.1038/s41598-020-67075-6

**Published:** 2020-06-19

**Authors:** QuanQiu Wang, Rong Xu

**Affiliations:** Center for Artificial Intelligence in Drug Discovery, School of Medicine, Case Western Reserve University, Cleveland Ohio, 44106 USA

**Keywords:** Literature mining, Microbiome

## Abstract

Many diseases are driven by gene-environment interactions. One important environmental factor is the metabolic output of human gut microbiota. A comprehensive catalog of human metabolites originated in microbes is critical for data-driven approaches to understand how microbial metabolism contributes to human health and diseases. Here we present a novel integrated approach to automatically extract and analyze microbial metabolites from 28 million published biomedical records. First, we classified 28,851,232 MEDLINE records into microbial metabolism-related or not. Second, candidate microbial metabolites were extracted from the classified texts. Third, we developed signal prioritization algorithms to further differentiate microbial metabolites from metabolites originated from other resources. Finally, we systematically analyzed the interactions between extracted microbial metabolites and human genes. A total of 11,846 metabolites were extracted from 28 million MEDLINE articles. The combined text classification and signal prioritization significantly enriched true positives among top: manual curation of top 100 metabolites showed a true precision of 0.55, representing a significant 38.3-fold enrichment as compared to the precision of 0.014 for baseline extraction. More importantly, 29% extracted microbial metabolites have not been captured by existing databases. We performed data-driven analysis of the interactions between the extracted microbial metabolite and human genetics. This study represents the first effort towards automatically extracting and prioritizing microbial metabolites from published biomedical literature, which can set a foundation for future tasks of microbial metabolite relationship extraction from literature and facilitate data-driven studies of how microbial metabolism contributes to human diseases.

## Introduction

Genetic, epigenetic, and environmental factors contribute to the susceptibility, progression and outcomes of many common complex diseases^1–3^. While significant progress has been made in understanding genetic, molecular, cellular aspects of human diseases, there is limited understanding how modifiable environmental factors such as food, nutrition, lifestyle, and physical activity are involved in human diseases and health. Human gut microbiota, an important modifiable intermediator between external environmental exposure and host genetics, exist in symbiotic relation with human hosts^4–8^. Accumulating biomedical evidence indicates that gut microbiota and their metabolites strongly influence disease susceptibility and progression in humans^9–12^. However, the underlying mechanisms remain largely unknown.

We have recently developed data-driven computational approaches to understand how microbial metabolites are mechanistically involved in various common complex diseases including colorectal cancer^13,14^, Alzheimer’s disease^15^, psoriasis^16^, and rheumatoid arthritis^17,18^. For example, we developed network-based systems approaches to examine genetic interactions between microbial metabolites and human diseases and revealed strong mechanistic links trimethylamine N-oxide (TMAO), a gut microbial metabolite of dietary meat and fat, and both colorectal cancer^13^ and Alzheimer’s disease^15^. These computationally generated findings were subsequently verified by other researchers using patient sample-based metabolomics studies, which showed that plasma TMAO is indeed positively associated with colorectal cancer risk^19^ and that the gut microbiota-derived metabolite TMAO is elevated in Alzheimer’s disease^20^.

In our previous data-driven studies of how microbial metabolites contribute to specific diseases, we used the 172 known microbial metabolites from the Human Metabolome Database (HMDB), the most comprehensive human metabolome database of over 114,100 small molecule metabolites found in the human body^21^. During our prior studies, we found that many microbial metabolites have been reported in biomedical literature, but not classified as microbial origin by HMDB, as shown in the sentence “*We further investigate the bioactivity of the confirmed metabolites, and identify two microbiota-generated metabolites (5-hydroxy-L-tryptophan and salicylate) as activators of the aryl hydrocarbon receptor*” (PMID 25411059). HMDB includes both 5-hydroxy-L-tryptophan (HMDB0000472) and salicylate (HMDB0001895), which means that these two microbial metabolites are found in human bodies. However, L-tryptophan is not classified as microbial metabolite but as a plant metabolite by HMDB. Consequently, our previous studies did not include L-tryptophan, though recent studies showed that microbial metabolite tryptophan may play vital roles in early life development, inflammatory bowel diseases and neurological diseases^22^. In order to systematically understand how gut microbial metabolism contributes to human disease and health, it is necessary to build a comprehensive list of microbial metabolites that are also found in human bodies. Metabolites found in human bodies can originate from a variety of different resources, including human hosts, gut bacteria, diet, plants, toxins, medications, among others. Microbial metabolites are defined as metabolites produced (not necessarily exclusively) by bacteria. In this study, we focus on metabolites that are not only produced by microbes/bacteria but also present in human bodies.

There are active research works in building software or knowledgebases related to human microbial metabolisms with the goal of facilitating developing computational models fto understand human and microbial metabolism. Medema *et al*. developed antiSMASH (antibiotics & Secondary Metabolite Analysis Shell), a comprehensive pipeline for genome-wide identification, annotation and analysis of secondary metabolite biosynthesis gene clusters in bacterial and fungal genomes^23^. Magnúsdóttir *et al*. developed AGORA (assembly of gut organisms through reconstruction and analysis) for genome-scale metabolic reconstructions based on literature-derived experimental data and comparative genomics^24^. Sung *et al*. developed a system-level framework of complex microbe–microbe and host–microbe chemical cross-talk and constructed a manually curated literature-based interspecies network of the human gut microbiota (NJS16)^25^. Noronha *et al*. constructed the Virtual Metabolic Human (VMH) database to capture information on human and gut microbial metabolism and links this information to hundreds of diseases and nutritional data. VMH was built by manually curating hundreds of genome-scale metabolic reconstructions^26^. Previous approaches reconstructed microbial metabolic activities based on automatically genome-wide reconstruction of s metabolite biosynthesis gene clusters in bacterial genomes^23,24^ or based on manual literature curation^25,26^. Leveraging evidence from tens of millions of published biomedical records, we are taking an alternative approach to automatically classify, extract and prioritize microbial metabolites from free-text documents.

The large number of published biomedical research articles is a rich resource of microbial studies. Automatically extracting machine-understandable knowledge of microbiome in human diseases is a challenging task^27^. Recently, researchers developed natural language processing and text mining techniques to extract disease-microbe (bacteria) relationship from published biomedical literature^28,29^. However, currently no research efforts have been devoted to extract microbial metabolites from biomedical literature. Our goal for this study is to extract microbial metabolites from free-text articles and differentiate metabolites of microbial origin from human metabolites originated in other sources (e.g., human hosts, plants, foods, toxins, pollutants, cosmetics, and drugs). We developed an integrated approach by combining text classification, named entity extraction (NER) and signal prioritization to automatically extract microbial metabolites from over 28 million MEDLINE records. Since the majority of published biomedical articles are not related to microbial studies, the important first step of our strategy was to find microbial metabolism-related articles (“*Text classification*”). We then performed dictionary-based extraction of metabolites from classified text documents (“*Named Entity Recognition*”). During out experiment, we found that the majority of metabolites extracted from microbial metabolism-related text documents are not microbial metabolites (i.e., originated in microbes). We then developed signal ranking algorithms to further differentiate microbial metabolites from metabolites of other origins (“*Signal prioritization*”). For algorithm evaluation, we manually curated top ranked metabolites. We analyzed the interactions between identified microbial metabolites and human genes, which may provide mechanistic insights into how gut microbial metabolism may contribute to human health.

To the best of our knowledge, our study represents the first effort towards large-scale extraction and prioritization of microbial metabolites from over 28 million published biomedical articles. Our study will set the foundation for future tasks of microbial metabolite entity recognition and relationship extractions. A comprehensive list of microbial metabolites will greatly facilitate data-driven studies of how gut microbial metabolism contributes to human health and diseases.

## Results

### Classification improves microbial metabolite extraction from MEDLINE articles

A total of 11,846 unique metabolites were extracted from all MEDLINE articles (see “Methods” section). The baseline NER approach, which was to extract microbial metabolites from all MEDLINE articles (no classification) has a precision of 0.014, recall of 0.959 and F1 of 0.028 (Table 1). The high recall of 0.959 demonstrates that MEDLINE is a comprehensive resource for microbial metabolites. The extremely low precision (0.014) may be due to: (1) the precision evaluated using the 172 known microbial metabolites from HMDB significantly under-estimated the true precision. As shown in our later manual curation, many true microbial metabolites are not captured by HMDB; and (2) extracting microbial metabolites from biomedical literature is a very challenging task. Microbial metabolites constitute only a small portion of all metabolites found in human body, therefore it is necessary to further differentiate microbial metabolites from metabolites originated in other sources such as human hosts, plants, foods, toxins, hosts, cosmetics and drugs.

**Table 1:** Overall performance of microbial metabolite extractions from unclassified MEDLINE records, classified microbial-related MEDLINE records, and classified microbial metabolism related MEDLINE records. 172 known microbial metabolites from HMDB were used as the gold standard. The 2017 HMDB was used.

Approach	Articles (n)	Extracted Metabolites (n)	Precision	Recall	F1
Baseline (Unclassified articles)	28,851,232	11,846	0.014	0.959	0.028
Classified - Microbial	42,431	2,346	0.049	0.674	0.092
Classified - Microbial Metabolism	16,728	2,016	0.055	0.640	0.101

By classifying MEDLINE articles into microbial-related (42,431 articles) (see “Methods” section), the performance of NER from classified texts as measured by F1 increased from 0.028 to 0.092, representing a 228% improvement; precision increased from 0.014 to 0.049, a 250% improvement; recall decreased from 0.959 to 0.674, a 29.7% decease. By further classifying MEDLINE articles into microbial metabolism-related (16,728 articles) (see “Methods” section), the F1 increased from 0.028 to 0.101, representing a 261% improvement; precision increased from 0.014 to 0.055, a 293% improvement; recall decreased from 0.959 to 0.640, a 33.2% decease (Table 1). We further compared the performance of NER (from 16,728 microbial metabolism-related articles) using metabolite lexicons constructed from the 2017 and 2018 versions of HMDB. Though the 2018 version of HMDB included significantly more metabolites than the 2017 version of HMDB, the precision and F1 are lower and the recalls are similar (precision = 0.44, recall = 0.651, F1 = 0.083) as compared to the performance based on the 2017 HMDB. In summary, microbial-related articles constitute only a small portion of all MEDLINE articles. Text classification before NER significantly improved microbial metabolite extraction from MEDLINE articles. Since microbial metabolites constitute only a small portion of all metabolites found in human bodies, the overall performance is still low, demonstrating the need for further prioritization of extracted metabolites.

### Prioritization further improves microbial metabolite extraction

**Figure 1:**
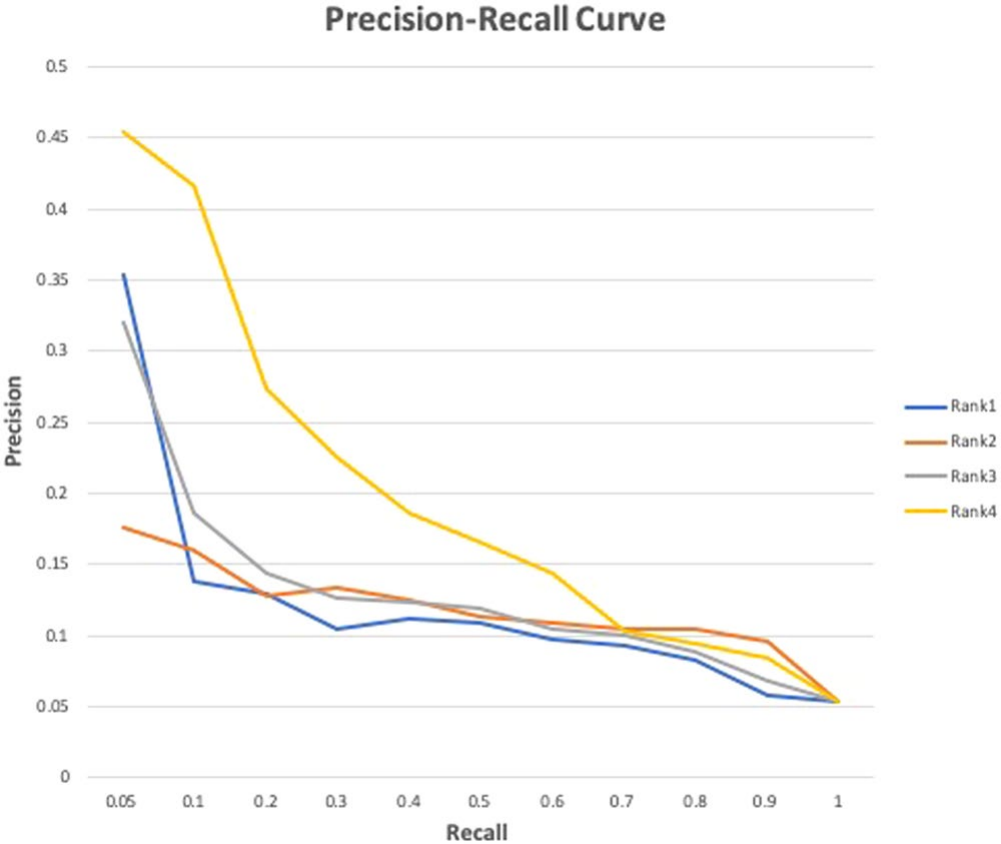
Precision-recall curves. Precisions and recalls were calculated using the 172 known microbial metabolites from HMDB as evaluation dataset. Extraction was performed on 16,728 classified microbial metabolism-related articles.

We developed four ranking algorithms to further prioritize metabolites extracted from the classified microbial metabolism-related articles (see “Methods” section). The four algorithms differ in how they emphasize the occurrence of a metabolite in classified documents and penalize its occurrence in all MEDLINE documents. Among the four ranking algorithms, $Ran{k}_{4}(m)$ is most effective in prioritizing known microbial metabolites among top (Fig. 1). The top ranked metabolites (at recall of 0.05) have a precision of 0.45, which represents a significant 741% increase as compared to the overall precision of 0.055 (at recall of 1.0). $Ran{k}_{2}(m)$ is the least effective because the occurrence of a metabolite in classified documents (often a small number) was overly penalized by its occurrence in all MEDLINE documents (often a big number). Note that the precisions were calculated using 172 known microbial metabolites from HMDB. We show in the next section that these precisions significantly underestimated the true precision. Here we only used them for algorithm comparison, not for true precision calculation.

### Many microbial metabolites have not been captured by HMDB

**Figure 2:**
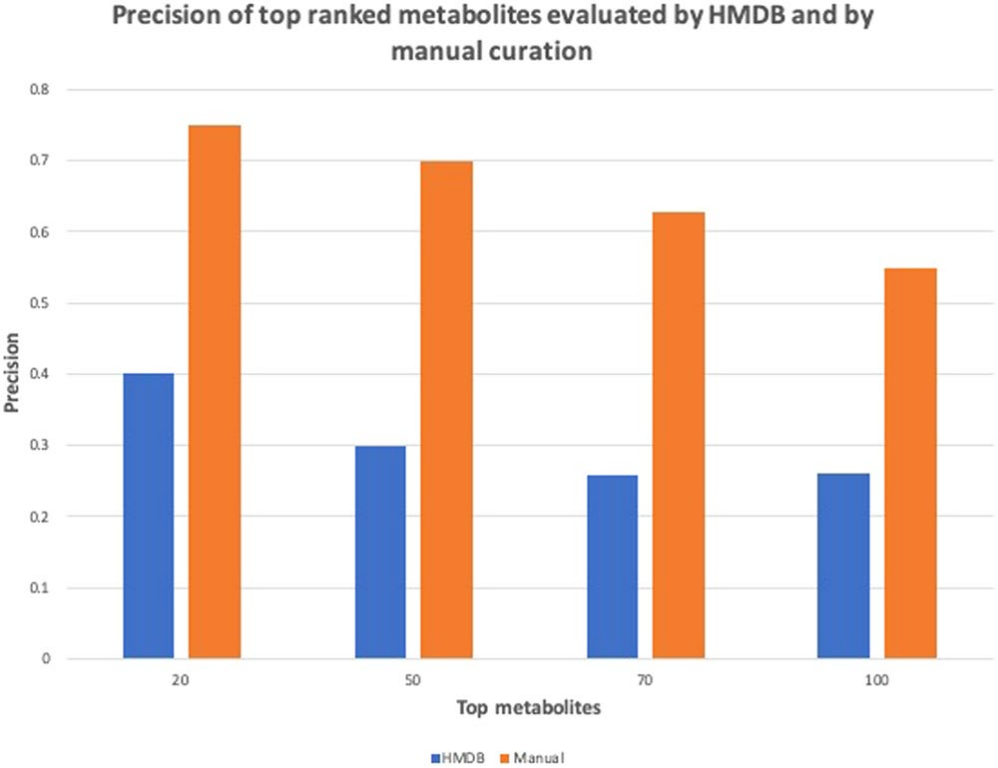
Estimated precisions (evaluated using 172 known microbial metabolites from HMDB) and true precisions (evaluated using the combined list of 201 microbial metabolites from HMDB and from manual curation) of top ranked metabolites at four ranking cut offs (top 20, 50, 70 and 100 metabolites).

Manual curation of top 100 ranked metabolites (see “Methods” section) identified 26 known microbial metabolites from HMDB and additional 29 new microbial metabolites. Note that these 29 metabolites are included HMDB but not classified as microbial metabolites. We compared estimated precisions (evaluated with 172 microbial metabolites from HMDB) and true precisions (evaluated with 172 from HMDB combined with the additional 29 from manual curation) at four ranking cutoffs (top 20, 50, 70 and 100 metabolites). As shown in Fig. 2, top ranked metabolites at each cutoff included many new microbial metabolites that have not yet captured in HMDB. For example, 15 of top 20 metabolites are true positives (true precision of 0.75), among which only 8 were captured by HMDB (precision of 0.4). Among top 100 metabolites, 55 are true positives (true precision of 0.55) and only 26 were included in HMDB (precision of 0.26). A total of 15 out of the top 20 metabolites has supporting evidence from either HMDB (8 metabolites) or biomedical literature (7 metabolites) (Table 2). In summary, our manual curation showed that as much as 29% microbial metabolites have not yet captured by HMDB. This result confirmed the importance of our effort in extracting additional microbial metabolites from biomedical literature. The fact that top ranked metabolites are highly enriched for true positives (true precision of 0.55) as compare to all extracted metabolites demonstrated the effectiveness of the prioritization algorithm (*Rank*_4_(*m*)).

**Table 2:** Top 20 ranked metabolites with supporting evidence from HMDB or biomedical literature (the unique identifier number used in PubMed or PMID shown). True positives (13 out of 20) are in bold. Metabolites without supporting evidence are denoted with “NO”.

Rank	Metabolite	Evidence	Rank	Metabolite	Evidence
1	**ellagic acid**	PMID 28616977	11	**trimethylamine**	HMDB
2	**butyric acid**	HMDB	12	**p-cresol sulfate**	HMDB
3	phenol-formaldehyde, cross-linked, tetraethylenepentamine activated	NO	13	**cholic acid**	PMID 26531326
4	**trimethylamine n-oxide**	HMDB	14	**ortho-hydroxyphenylacetic acid**	HMDB
5	chymosin preparation, Escherichia coli k-12	NO	15	**l-lactic acid**	PMID 30189365
6	**propionic acid**	HMDB	16	**indoxyl sulfate**	HMDB
7	**urolithin b**	PMID 19716282	17	gonyautoxin v	NO
8	**indoxyl**	PMID 30029499	18	**acetic acid**	HMDB
9	trans-aconitic acid	NO	19	inulobiose	NO
10	urolithin c	PMID 19716282	20	urolithin d	PMID 19716282

### Analysis of microbial metabolite-gene interactions

We analyzed the 201 microbial metabolites (172 from HMDB and 29 additional from manual curation) in order to identify human genes commonly regulated by gut microbial metabolites (see “Methods” section). A total of 11,201 human genes are associated with at least one of these 201 microbial metabolites (details described in the Method Section under the subsection “**Systematic analysis of gut microbial metabolite-host genetic interactions**”). These genes were then ranked by the number of associated microbial metabolites. The top 20 genes are shown in Table 3. These top genes are mainly involved in metabolism of amino acids (e.g., ACACA, ACACB, DPYD, F2, GLUL, GSR, SLC25A21, TYR), nucleotides (e.g., CAT, DPYD, GSR, UMPS, UPP2), fatty acids (e.g., ACACA, ACACB, DECR1, DPYD) and glucose (e.g., GUSB, SORD, TALDO1).

**Table 3:** Top 20 human genes ranked by the number of associated microbial metabolites.

Symbol	Name	Score	Symbol	Name	Score
TMPRSS11D	transmembrane serine protease 11D	53	TSPO	translocator protein	30
CAT	Catalase	50	ODC1	ornithine decarboxylase 1	30
ALB	albumin	47	ACACA	acetyl-CoA carboxylase alpha	28
DECR1	2,4-dienoyl-CoA reductase 1	44	GSR	glutathione-disul_de reductase 29	27
UMPS	Uridine Monophosphate Synthetase)	32	ACACB	acetyl-CoA carboxylase beta	27
TALDO1	transaldolase 1	31	F2	coagulation factor II, thrombin	26
GLUL	glutamate-ammonia ligase	31	DPYD	dihydropyrimidine dehydrogenase	26
GLUL	glutamate-ammonia ligase	31	TYR	tyrosinase	26
PRDM10	PR/SET domain 10	31	UPP2	uridine phosphorylase 2	26
GUSB	glucuronidase beta	30	SORD	sorbitol dehydrogenase	25

The top ranked two gene TMPRSS11D (transmembrane serine protease 11D) and CAT (catalase) is associated with 53 and 50 of the 201 microbial metabolites, respectively. TMPRSS11D may play biological roles in the host defense system on the mucous membrane and is involved in hearing loss and airway inflammation^30^. Catalase is an important enzyme in protecting the cell from oxidative damage by reactive oxygen species (ROS). It catalyzes hydrogen peroxide, a harmful by-product of many normal metabolic processes, into water and oxygen^31^. Genetic studies showed that catalase is involved in metabolic syndromes, including obesity, type 2 diabetes, atherosclerosis, hyperglycemia, dyslipidemia, and hypertension^32–34^. Recent studies showed the dysbiosis of gut microbiota is associated with obesity^35^, type 2 diabetes^36^, atherosclerosis^37^, hyperglycemia^38^, dyslipidemia^39^, and hypertension^40^. Our analysis of the interaction between microbial metabolites and human genetics provides possible mechanistic links between gut microbiota and diseases (e.g., gut microbiota = > type 2 diabetes). For example, butyric acid is one of the three short-chain fatty acids (SCFAs) that are pivotal in human nutrient acquisition, immune function, cell signaling, proliferation control and pathogen protection [Kohl]. Butyric acid is associated with CAT based on the chemical associations from the STITCH database^41^. One immediate hypothesis can be generated is that human gut microbiota contribute to type 2 diabetes by producing butyric acid in digestion of dietary fiber, which can then target catalase gene/protein in human body. We made all the results publicly available and hope that our results may set the foundation for biomedical researchers to conduct hypothesis-driven mechanistic studies of how gut microbial metabolites interact with host genetics in contributing to human health and diseases.

## Discussion

We developed an integrated approach by combining text classification, named entity extraction (NER) and signal prioritization to automatically extract microbial metabolites from over 28 million MEDLINE records. We then performed data-driven analysis of the interactions between microbial metabolite and human genes. Our study represents the first effort towards large-scale extraction and prioritization of microbial metabolites from published biomedical articles. A few limitations warrant further discussion.

In this study, we used the fields of Title, Abstract, Mesh Headings, Keywords, and Chemicals of MEDLINE records for both text classification and named entity recognition. While important findings are often captured in these fields, it is likely that some microbial metabolites are listed in full-text fields, including embedded tables or even supplementary data. In our future works, we will further improve our study by using both MEDLINE records and the collection of the Open Access Subset of full-text articles via PMC (https://www.ncbi.nlm.nih.gov/pmc/tools/textmining).

The signal prioritization algorithms prioritize metabolites based on their differential distributions in microbial metabolism-specific MEDLINE records versus all MEDLINE records. This approach ranks highly those highly microbial specific metabolites such as trimethylamine N-oxide. However, it has limited performance in ranking common microbial metabolites such as “*hydrogen*”, a gas whose biological production has only been found in microorganisms. The reason is that the term “*hydrogen*” commonly appears in both microbial metabolism-specific and non-microbial metabolism-specific MEDLINE records. When it appears in microbial metabolism specific MEDLINE records, it often refers the microbial metabolite “*hydrogen*”. However, the term “*hydrogen*” also frequently appears in non-microbial metabolism-specific MEDLINE records, where it often does not refer to microbial metabolite. While our study showed the effectiveness of the prioritization algorithms in prioritizing microbial specific metabolites such as trimethylamine N-oxide and butyric acid, there is space for further improvements in prioritizing non-specific microbial metabolites such as *hydrogen*.

By classifying MEDLINE articles into microbial-related, the F1 increased by 228% and the precision increased by 250%. However, the recall decreased from 0.959 to 0.674, a 29.7% drop, demonstrating that some microbial metabolites indeed appeared in the fields of MEDLINE articles, but the text classification may have missed those articles. One strategy to improve the classification of MEDLINE records is to expand the list of classification keywords (e.g., *microbiota, microbiome*) by including specific bacteria species names (e.g., *Escherichia coli*, *Clostridium spp*. and *Bacteroides spp*). If a record contains a name of bacteria/microbe, it will be classified as microbial-related.

Our main goal of this study was to identify metabolites that are included in HMDB but not classified as microbial origin. Our manual curation demonstrated that as many as 29% of microbial metabolites were included in HMDB but not classified as being originated in microbes. However, during our experiment, we found that some microbial metabolites were clearly stated in biomedical literature, but the names were not included in HMDB. For example, the sentence “*Marked associations between bacterial species (Clostridium genus) and the amount of some metabolites were identified*. *Moreover*, *trans-resveratrol and resveratrol*-*derived* ***microbial metabolites (dihydroresveratrol*** and ***lunularin)*** were also identified” (PMID 26156396) contains two microbial metabolites *dihydroresveratrol* and *lunularin*. None of these two microbial metabolites are included in HMDB, therefore not included in our HMDB-based lexicon and missed from the dictionary-based NER. In our future studies, we will perform de-novo NER from biomedical literature. In our previous studies, we developed pattern-based iterative learning approaches for *de-novo* NER (e.g., diseases and drugs)^42,43^ and relationship extraction (e.g., disease-phenotype, disease-risk factor)^44–46^. In future studies, we will complement this study by developing iterative learning approaches to extract additional microbial metabolites from MEDLINE records.

Our future directions also include metabolite-bacteria and microbial metabolite-disease relationship extractions. Currently, the Unified Medical Language System (UMLS) Metathesaurus contains 383,775 unique concepts of bacterium and 106,786 unique concepts of diseases or syndromes^47^. In this study, we have manually curated top 100 prioritized metabolites and obtained 29 new microbial metabolites. In order to build a comprehensive and accurate lexicon of microbial metabolites, we will need manually curate more top ranked metabolites. Manual curation is a labor-intensive work. The text classification, subsequent NER from classified texts, and signal prioritization will significantly reduce our future manual curation effort by significantly enriching true signals among top ranked metabolites.

## Conclusion

Our study represents the first effort towards large-scale extraction and prioritization of microbial metabolites from over 28 million published biomedical articles. We analyzed the interactions between identified microbial metabolites and human genes, which may provide mechanistic insights into how gut microbiota contribute to human health and diseases. Our study will set the foundation for future microbial metabolite entity recognition and relationship extraction. A comprehensive list of microbial metabolites will also greatly facilitate data-driven studies of how gut microbial metabolites interact with host genetics in different human diseases. The identification of microbial metabolites and the understanding of their role as key mediators through which these bacteria are involved in disease pathogenesis will provide insight into the molecular mechanisms of human health and diseases and enable new possibilities for disease diagnosis, prevention, and treatment.

## Methods

### Text classification

We downloaded a total of 28,851,232 MEDLINE records (published up to July, 2018) from the National Library of Medicine (https://www.nlm.nih.gov/databases/download/pubmed_medline.html. MEDLINE fields of Title, Abstract, Mesh Headings, Keywords, and Ch*emicals* were used for both text classification and named entity recognition. Since not all the 28,851,232 MEDLINE records are related to microbial studies, we classified MEDLINE records to find microbial-related articles in order to improve subsequent microbial metabolites extractions from MEDLINE records. Instead of using standard supervised machine-learning approaches for text classification that often require a large amount of manually annotated training data, we used an intuitive approach that simulates how researchers perform meta-analysis and systematic review of microbial studies. In fact, researchers performed systematic review of microbial studies and obtained articles by searching for “(*microbiome* | *microbiota* | *microflora*)” on PubMed. We used these typical search terms (“*microbial*”, “*microbiome*”, “*microbiota*”, “*microflora*” and “*microbial*”) as features to classify and obtain microbial-related text documents from all MEDLINE records. All the fields of MEDLINE records (*Title*, *Abstract*, *Mesh Headings*, *Chemicals*, *Keywords*) were used for text classification. Since the majority of microbial studies are not related to microbial metabolism, we further classified microbial-related articles into metabolism-related using metabolism-related keywords (“*metabolism*”, “*metabolite*”, “*metabolic*” and “*metabolome*”). Our comparison analysis showed that the performance of NER from classified microbial metabolism-related articles was further improved as compared to that from microbial-related articles.

### Named entity recognition and evaluation

#### Named entity recognition

We performed dictionary-based named entity recognition to extract microbial metabolites from MEDLINE records. We first built a lexicon that consists of all metabolites from HMDB^21^, including preferred names and their synonyms. The lexicon consists unique metabolite concepts (e.g., butyric acid) and their corresponding synonyms (e.g., *1-butanoate*, *1-butanoic acid*, *butanoic acid*, *butyrate*, *ethylacetate*, *kyselina maselna*, *propanecarboxylate*, *propylformate* and *propylformic acid*). We experimented with both year 2017 and year 2018 versions of HMDB. The lexicon based on the 2017 version comprised of 42,003 unique metabolite concepts, 188,153 synonyms and 365,632 synonym = > concept mappings. The lexicon based on the 2018 version is significantly larger and contains 114,100 unique metabolites, 510,603 synonyms and 11,131,600 synonym = > concept mappings. The HMDB-based lexicon of human metabolites was used to recognize metabolites and their synonyms from fields of MEDLINE records (*Title*, *Abstract*, *Mesh Headings*, *Chemicals*, *Keywords*). Extracted entities were then normalized by mapping synonyms to their preferred names (e.g., *butyrate* = > *butyric acid*, *propylformate* = > *butyric acid*). For comparison, we performed NER from (1) all MEDLINE records (baseline), (2) classified microbial-related text documents; and (3) classified microbial metabolism-related text documents. We also experimented NER based on lexicons constructed from 2017 and 2018 versions of HMDB. Based on our experiments and comparison results showing that lexicon based on 2017 version had both higher precision and coverage than the 2018 version, our subsequent experiments were based on the 2017 version.

#### Evaluation

We evaluated the performance of the NER approaches using the 172 known microbial metabolites from HMDB. HMDB is the most comprehensive human metabolome database of over 114,100 small molecule metabolites found in the human body. The 172 classified microbial metabolites captured in HMDB are currently the best-known list of human metabolites that are originated in microbes. We previously developed data-driven computational approaches to understand how microbial metabolites are mechanistically involved in various common complex diseases using this list of 172 microbial metabolites^13–18^. Standard measures of precision (fraction of recognized entities as positive that are truly positive), recall (true positive rate) and F1 (harmonic average of the precision and recall) were calculated and compared. Since many microbial metabolites are reported in literature but not captured by the list of 172 microbial metabolites from HMDB (as shown later in our study demonstrating that as much 29% of top 100 extracted microbial metabolites were not captured by the list of 172 microbial metabolites), the calculated precision significantly under-estimated the true precision. Therefore, these 172 known microbial metabolites were used to compare relative performances among different approaches but not for true precision calculation.

### Signal prioritization and evaluation

#### Prioritization

The majority of metabolites extracted from the classified microbial metabolism-related MEDLINE records were not microbial metabolites. We further prioritized the extracted metabolites in order to differentiate metabolites originated from microbes from metabolites originated from other resources (e.g., hosts, plants, foods, pollutants, and drugs). We hypothesized that if a metabolite is significantly enriched in the classified microbial metabolism-related MEDLINE records as compared to all MEDLINE records, it is more likely a microbial metabolite.

An effective prioritization algorithm will rank highly those true positive microbial metabolites and enrich them among top. Based on the prioritization scores, if a metabolite has higher score than others, it means that it is more likely to be classified as a microbial metabolite. The performance of different ranking algorithms was evaluated and compared using the standard ranked precision-recall curves^48,49^, where precision and recall measures at different cutoffs of ranking scores.

$$Ran{k}_{1}(m)=d{f}_{c}(m)$$

where $d{f}_{c}(m)$ represents the number of classified MEDLINE articles/documents in which a metabolite appeared. $Ran{k}_{1}(m)$ is a baseline ranking algorithm and ranks a metabolite (m) based on the its frequency in classified documents ($d{f}_{c}(m)$).

$$Ran{k}_{2}(m)=\frac{d{f}_{c}(m)}{df(m)-d{f}_{c}(m)}$$

$Ran{k}_{2}(m)$ offsets a metabolite’s occurrence in classified microbial-related documents by its occurrence in non-microbial related articles. $d{f}_{c}(m)$ is the number of classified MEDLINE documents in which a metabolite *m* appeared, $df(m)$ is the metabolite’s occurrence in all MEDLINE records, and $df(m)-d{f}_{c}(m)$ is its occurrence in non-microbial related articles.

$$Ran{k}_{3}(m)=\frac{d{f}_{c}(m)}{\mathrm{ln}(df(m)-d{f}_{c}(m))}$$

$Ran{k}_{3}(m)$ is similar to $Ran{k}_{2}(m)$ except that a metabolite’s occurrence in microbial-related documents was offset in less degree by its occurrence in non-microbial-related articles.

$$Ran{k}_{4}(m)=\frac{d{f}_{c}(m)\ast d{f}_{c}(m)}{df(m)-d{f}_{c}(m)}$$

$Ran{k}_{4}(m)$ is similar to $Ran{k}_{2}(m)$ except that the signal was further boosted by a metabolite’s occurrence in microbial-related documents. Our results showed that $Ran{k}_{4}(m)$ was the most effective among four approaches. This is consistent with our hypothesis that if a metabolite is highly enriched in the classified microbial metabolism-related MEDLINE records (represented as the numerator $d{f}_{c}(m)\ast d{f}_{c}(m))$ compared to all MEDLINE records (represented as the denominator $df(m)-d{f}_{c}(m))$, it is more likely a microbial metabolite. $Ran{k}_{4}(m)$ is more effective than the other three methods because it further boosted the signal of a metabolite’s presence in microbial-related documents ($d{f}_{c}(m)\ast d{f}_{c}(m))$ while at the same time penalized its appearance in the whole MEDLINE. The reason in choosing square function to boost the signal of metabolite’s presence in microbial-related documents is that when other higher-order polynomial or exponential functions are used, the numerator will quickly become too big and overpower the denominator. However, our ranking algorithm is to prioritize terms that appear often in classified documents but at the same time penalize terms that also appear very often in all MEDLINE records.

#### Evaluation

We used Precision-Recall (PR) curves to evaluate and compare four prioritization methods. PR curves are often used to evaluate ranked results in information retrieval and classification^48,49^. A PR space is defined as precision and recall as *x* and *y* axes, respectively. Using the 172 microbial metabolites from HMDB as the evaluation dataset, we calculated precisions at 11 different recall cutoffs (0.05, 0.1, 0.2, … 1.0) and plotted the PR curves to compare the four ranking algorithms.

### Manual curation and evaluation

We manually curated top 100 metabolites (ranked by $Ran{k}_{4}(m)$). For each of the top 100 metabolites, we retrieved all MEDLINE articles and corresponding PubMed ID (PMIDs) where it appeared. Two authors manually read all these articles independently to decide if a metabolite was indeed a microbial metabolite. If a metabolite was determined by both curators as microbial origin, then it was classified as microbial metabolite. Manual curation data (the manually classified microbial metabolites and the PMIDs where there is evidence supporting their classifications) along with other data is publicly available at https://github.com/qxw5/microbiome_metabolites_nlp/tree/master/data. We obtained a total of 201 microbial metabolites by combining 172 known microbial metabolites from HMDB with the additional 29 new microbial metabolites manually curated from the top 100 metabolites. The true precision of the top 100 metabolites were then calculated using the 201 microbial metabolites. We then evaluated the effectiveness of $Ran{k}_{4}(m)$ by calculating the true precisions at four ranking cutoffs (top 20, 50, 70 and 100 metabolites).

### Systematic analysis of gut microbial metabolite-host genetic interactions

We analyzed the interactions between human genes and each of the 201 microbial metabolites. Genes associated with microbial metabolites were obtained from the STITCH (Search Tool for Interactions of chemicals) database^50^. The STITCH database contains data on the interactions between 500,000 small molecules and 9.6 million proteins from 2,031 organisms. In this study, we used chemical-gene associations found in human body, which include 94,473,339 chemical-gene pairs for 473,043 chemicals and 19,121 human genes (data accessed in July, 2018). Among the 201 microbial metabolites, 150 were mapped to chemical names from the STITCH database. For example, we mapped butyric acid from HMDB to butyrate in STITCH and obtained a total of 815 butyrate-associated genes. Each human gene was then ranked based on the number of associated microbial metabolites. For example, the gene “CAT” is associated with 50 out of the 150 mapped microbial metabolites and has a ranking score of 50. Gene “LIN28A” is associated with only one microbial metabolite and has a ranking score of 1. The goal of this analysis is to identify host genes commonly regulated by gut microbial metabolites.

## Data Availability

Project name: Microbiome Metabolites NLP. Project home page: https://github.com/qxw5/microbiome_metabolites_nlp. Operating system(s): Platform independent. Programming language: Java (>1.5). License: MIT License. The data set(s) supporting the results of this article are available at https://github.com/qxw5/microbiome_metabolites_nlp/tree/master/data.
